# Mouse Subcutaneous BCG Vaccination and Mycobacterium tuberculosis Infection Alter the Lung and Gut Microbiota

**DOI:** 10.1128/spectrum.01693-21

**Published:** 2022-06-02

**Authors:** Fabiola Silva, Raphaël Enaud, Elizabeth Creissen, Marcela Henao-Tamayo, Laurence Delhaes, Angelo Izzo

**Affiliations:** a Department of Microbiology, Immunology and Pathology, Colorado State University, Fort Collins, Colorado, USA; b CHU de Bordeaux, CRCM Pédiatrique, CIC 1401, Bordeaux, France; c Université de Bordeaux, Centre de Recherche Cardio-Thoracique de Bordeaux, U1045, Bordeaux, France; d CHU de Bordeaux: Laboratoire de Parasitologie-Mycologie, Université de Bordeaux, Bordeaux, France; e Centenary Institute, University of Sydney, Sydney, Australia; Quest Diagnostics Nichols Institute

**Keywords:** BCG vaccine, tuberculosis, lung microbiota, gut microbiota, gut–lung axis

## Abstract

The objective of this study was to characterize the effect of Bacillus Calmette-Guérin (BCG) vaccination and M. tuberculosis infection on gut and lung microbiota of C57BL/6 mice, a well-characterized mouse model of tuberculosis. BCG vaccination and infection with M. tuberculosis altered the relative abundance of Firmicutes and Bacteroidetes phyla in the lung compared with control group. Vaccination and infection changed the alpha- and beta-diversity in both the gut and the lung. However, lung diversity was the most affected organ after BCG vaccination and M. tuberculosis infection. Focusing on the gut–lung axis, a multivariate regression approach was used to compare profile evolution of gut and lung microbiota. More genera have modified relative abundances associated with BCG vaccination status at gut level compared with lung. Conversely, genera with modified relative abundances associated with M. tuberculosis infection were numerous at lung level. These results indicated that the host local response against infection impacted the whole microbial flora, while the immune response after vaccination modified mainly the gut microbiota. This study showed that a subcutaneous vaccination with a live attenuated microorganism induced both gut and lung dysbiosis that may play a key role in the immunopathogenesis of tuberculosis.

**IMPORTANCE** The microbial communities in gut and lung are important players that may modulate the immunity against tuberculosis or other infections as well as impact the vaccine efficacy. We discovered that vaccination through the subcutaneous route affect the composition of gut and lung bacteria, and this might influence susceptibility and defense mechanisms against tuberculosis. Through these studies, we can identify microbial communities that can be manipulated to improve vaccine response and develop treatment adjuvants.

## INTRODUCTION

Tuberculosis (TB) is a world-leading infectious disease caused by Mycobacterium tuberculosis. According to the World Health Organization (WHO) Global Tuberculosis Report 2019, about 10 million people developed TB, and 1.4 million died of the disease (1). In humans, TB mainly affects the lower respiratory tract. When infection occurs, in most cases, the bacterium is contained by the host immune response as a latent infection (2, 3). However, if coinfection or immune suppression occurs, the individual develops the active and infectious phase of the disease (4). The only vaccine available against TB is the Bacillus Calmette-Guérin (BCG), a live attenuated strain of Mycobacterium bovis. Nevertheless, this vaccine has a variable degree of protection (4–6). The high variability of protection has been attributed to BCG strain variants, previous exposure to environmental mycobacteria, genetic variability on immune responses, and nutritional status (7, 8). Currently, the development of a new and effective vaccine has had limited success. This can be explained, to some extent, by the lack of knowledge of several key aspects in the immunopathogenesis of the disease. In addition, the contribution of the local microbiota to the disease progression has not been elucidated.

The gut microbiota and its impact on infectious and noninfectious diseases is currently an intense area of research (9–12). For instance, in the gut alone, microbial genes are hundreds of times more abundant than human genes, and they form a complex network within eukaryotic cells (13). The gut microbiota is critical for the development and homeostasis of the immune system, immune tolerance, catabolism of dietary fibers, and biosynthesis of amino acids and neuroactive amines (14). Thus, the innate immune system and gut microbiota affect one another through complex interactions and pathways, and this cross talk has been determined to be crucial for human health (15). The gut microbiota directly impacts the lung immune response, a communication known as the gut–lung axis (16–18). For instance, lung infection severity has been correlated with gut dysbiosis (19). This interaction has implications in the lung capacity to control allergies and infectious diseases (20). Short-chain fatty acids (SCFAs) produced by gut bacterial metabolism can travel through the bloodstream and induce immune cell development in the bone marrow and influence lung immune responses (14, 21, 22). Similarly, cells can migrate from the gut (e.g., innate lymphoid cells and T helper 17 cells) to the lung and impact the local immunity (23). In addition, metabolic and infectious diseases along with the use of antibiotics trigger gut dysbiosis, which is linked with an impaired immune response in the lung (24).

In the past decade, the lung was considered a sterile organ (25). Consequently, the U.S. National Institute of Health initially neglected this organ in the Human Microbiome Project (26–28). However, later it was revealed that the lung has a unique and complex microbiota worthy of analysis, whose composition and biomass are governed by its physiology. Moreover, this organ is constantly exposed to microorganisms, and its composition and biomass is influenced by microbial inhalation, salivary micro-aspiration, cough, local immunity, and mucociliary clearance (29). Other considerations include that the lung microenvironment has unique characteristics, such as an increased CO_2_ and low O_2_ partial pressure, elevated surface area, and higher temperature than the upper respiratory tract (29, 30). Some diseases such as asthma, chronic obstructive pulmonary disease (COPD), and cystic fibrosis (CF), have been associated with an imbalance of specific phyla and genera within the lung (31–33). On the other hand, not enough is known about the impact that BCG vaccination and M. tuberculosis infection may have on the gut and lung microbiota. Comparing gut and lung microbiota shifts may open new aspects of TB disease dynamics and explore how a subcutaneous vaccination may impact these two organs and the gut–lung axis. Exploring these questions would be essential to addressing the potential microbiota changes that BCG vaccination and M. tuberculosis infection cause in the host. Therefore, the characterization of the gut and lung microbiota will be useful to detect disease markers, to associate gut and lung dysbiosis with common infectious and noninfectious diseases, and to contribute to the development of new therapeutics. The objective of this study was to establish gut and lung microbiota features in C57BL/6 mice, after BCG vaccination and M. tuberculosis infection, and to analyze the gut and lung microbiota to determine if specific microbial genus were affected by subcutaneous vaccination and pulmonary infection.

## RESULTS

### BCG vaccination and M. tuberculosis infection changed the alpha-diversity in the gut and lung; however, BCG vaccination had a more pronounced effect on lung diversity.

**FIG 1:**
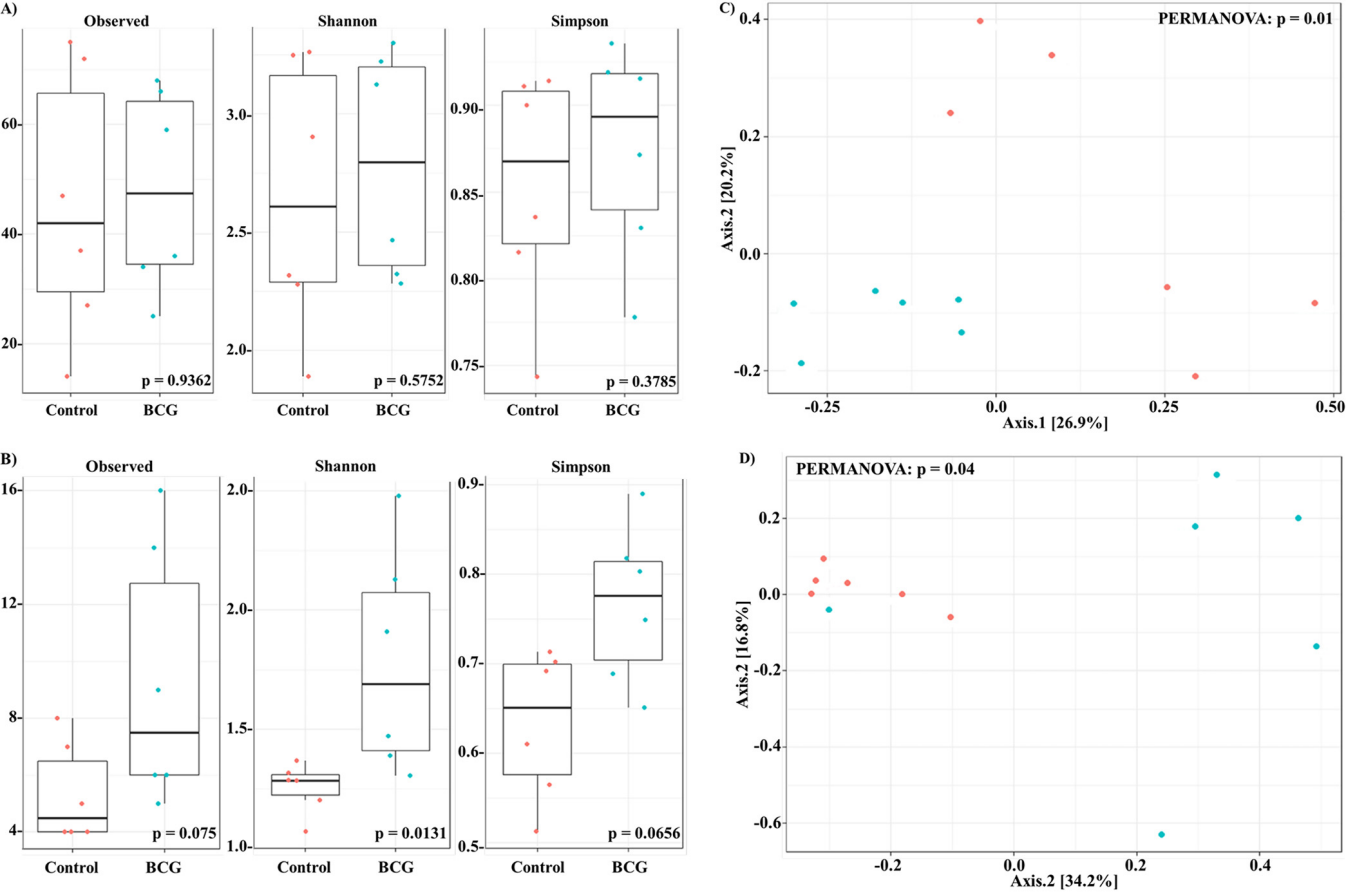
Microbiota alpha-and beta-diversity of gut and lung samples from control and BCG groups. Diversity metrics from gut (A, C) and lung (B, D) samples. Alpha-diversity plots shown in A and B. Beta-diversity clustering shown in C and D. Analyses based control (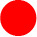) *vs* BCG groups (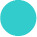).

To analyze the vaccination effect, uninfected mice (control and BCG groups) were divided into two groups (*n* = 6 per group) independent of day of sacrifice (7 or 21). Observed Amplicon Sequence Variants (ASVs), Shannon, and Simpson indexes were used to determine differences within alpha-diversity of gut and lung. Whereas in the gut, no statistical significance was observed (Fig. 1), in the lung the control group was statistically less diverse than the BCG group (Fig. 1 and *P*-values at 0.0131 of Shannon indexes).

**FIG 2:**
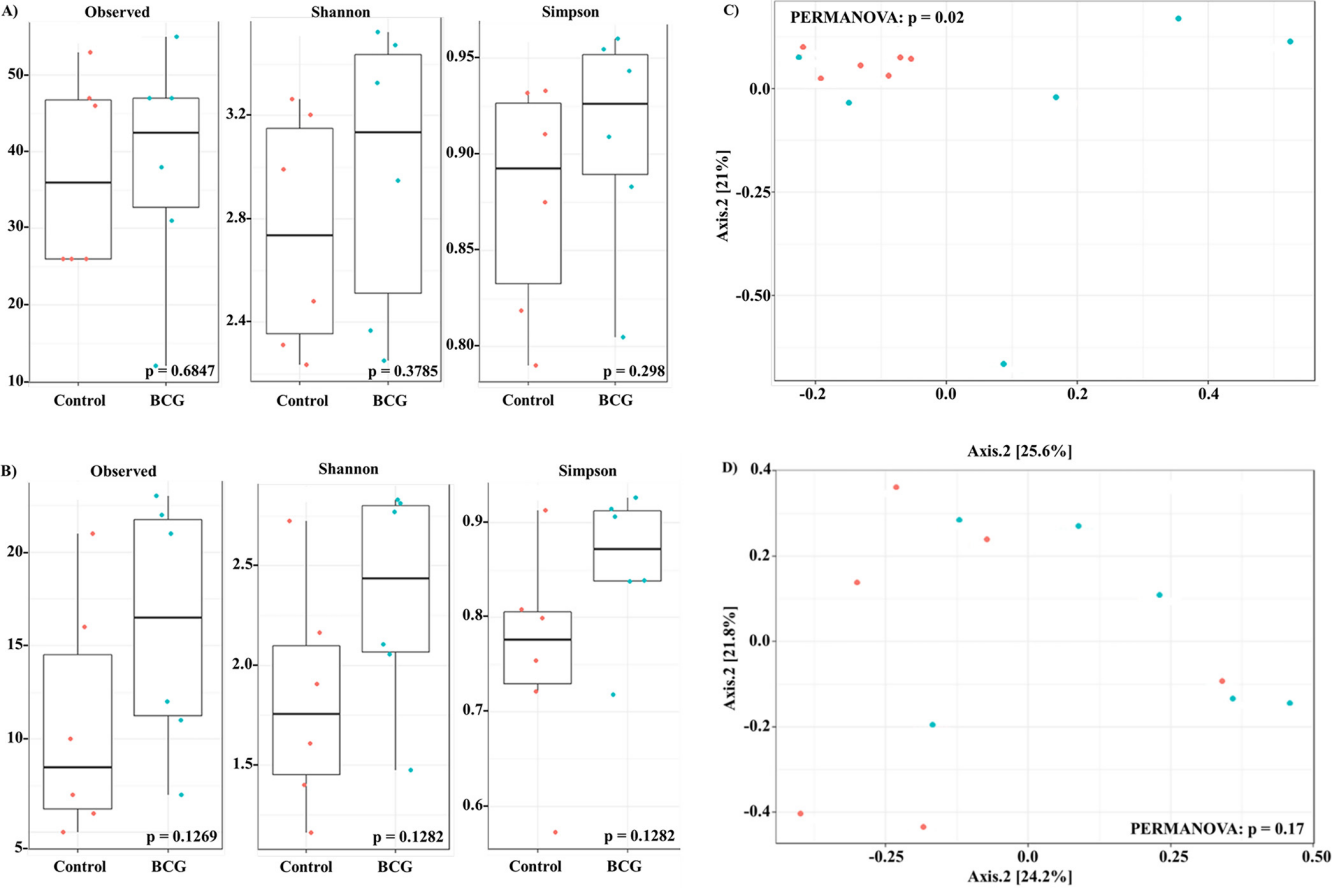
Microbiota alpha-and beta-diversity of gut and lung samples from BCG and BCG+Mtb groups. Diversity metrics from gut (A, C) and lung (B, D) samples. Alpha-diversity plots shown in A and B. Beta-diversity clustering shown in C and D. Analyses based on infected and vaccinated status of the mice. BCG (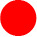) *vs* BCG+Mtb group (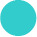).

**FIG 3:**
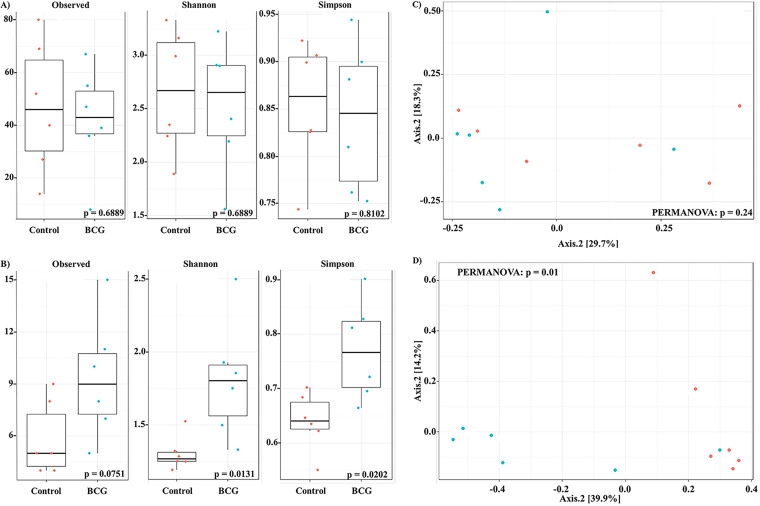
Microbiota alpha-and beta-diversity of gut and lung samples from control and Mtb groups. Diversity metrics from gut (A, C) and lung (B, D) samples. Alpha-diversity plots shown in A and B. Beta-diversity clustering shown in C and D. Analyses based on infected unvaccinated mice. control (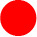) *vs* Mtb group (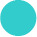).

To analyze the effect of M. tuberculosis infection in the gut and lung microbiota, infected and not infected mice were divided into two groups (*n* = 6 per group) independent of day of sacrifice (7 or 21). When comparing the BCG with BCG+Mtb groups, a statistical significance was not observed in the alpha-diversity measures at the gut or the lung levels (Fig. 2A and B, respectively). However, when the control with Mtb groups were compared, the Mtb group showed a higher alpha-diversity in the lungs (Fig. 3B and *P*-values at 0.0131 and 0.0202 of Shannon and Simpson indexes, respectively).

### Gut and lung beta-diversity confirmed separation between groups depending on BCG vaccination or M. tuberculosis infection.

At the beta-diversity level, BCG group was in closer proximity in the gut and the lung, which indicated similar gut and lung microbial community composition (Fig. 1; permutational multivariate ANOVA [PERMANOVA] *P* = 0.01 and 0.04, respectively). This clustering according to BCG vaccination was then confirmed when analyzing the BCG with BCG+Mtb groups at the gut level (Fig. 2C; PERMANOVA *P* = 0.02).

At the lung level, we confirmed the effect of M. tuberculosis infection on alpha-diversity (Fig. 3B). As samples obtained from the Mtb group from day 7 and day 21 postinfection indicated a highly similar lung microbial community composition (Fig. 3D; PERMANOVA *P* = 0.01).

### Firmicutes, Bacteroidetes, and Proteobacteria abundances were the most affected phyla after BCG vaccination and M. tuberculosis infection.

**FIG 4:**
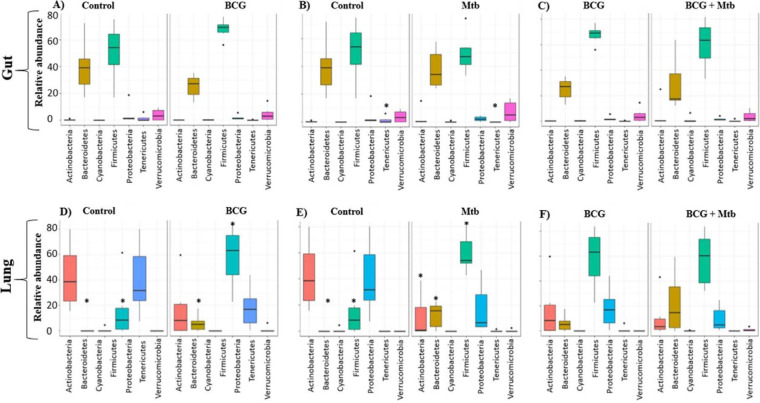
Distribution of phylum relative abundance caused by vaccination and infection. Vaccination and infection effects on gut (A–C) and lung (D–F). control *vs* BCG groups (A, D), control *vs* Mtb groups (B, E), BCG *vs* BCG+Mtb groups (C, F). Significant at *P* < 0.05, * using Wilcoxon rank sum test with continuity correction are reported among Actinobacteria 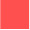, Bacteroidetes 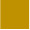, Cyanobacteria 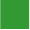, Firmicutes 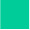, Proteobacteria 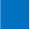, Tenericutes 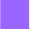, and Verrucomicrobia 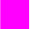.

At the gut level, the BCG and Mtb groups showed a tendency to increase the relative abundance of Firmicutes and Bacteroidetes (Fig. 4A and B), and only the Tenericutes phylum was significantly decreased in the Mtb group (Fig. 4B). At the lung level, there were significant differences in the relative abundance of and Bacteroidetes in the BCG and Mtb groups compared with the control group (Fig. 4D and E). In addition, Proteobacteria phylum appeared in higher proportion in the control group (Fig. 4D), and it was significantly decreased in Mtb group (Fig. 4E).

**FIG 5:**
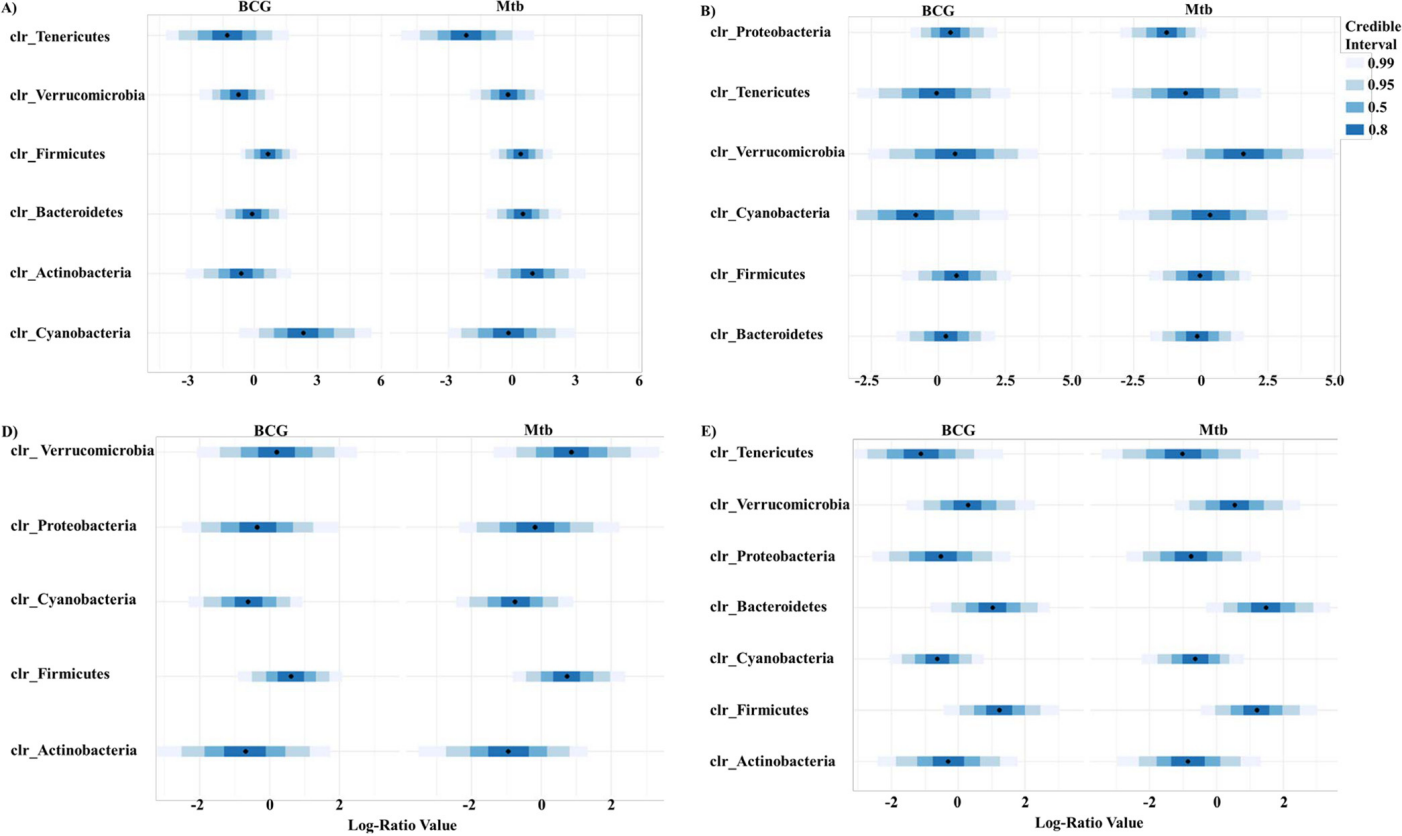
Credible intervals of phyla associated with the BCG vaccination and M. tuberculosis infection. Phyla found to be credibly associated with BCG vaccination and M. tuberculosis infection status on gut (A, B) and lung (C, D) in at least one of our two conditions at day 7 (A, C) and day 21 (B, D) using a multivariate regression model. Intervals that do not include 0 are determined associated with the studied factor.

These results were confirmed using a multivariate regression model previously applied to microbiota analysis (34). Firmicutes relative abundance in the gut was positively correlated with BCG vaccination and M. tuberculosis infection at day 7 (Fig. 5A) and with BCG vaccination at day 21 (Fig. 5B). A similar positive correlation was observed in the lung at both times (Fig. 5C and D). These results could have some association with the protective immune response induced by BCG in M. tuberculosis-infected mice. On the other hand, no statistical difference was observed in the abundance of Bacteroidetes or Proteobacteria when comparing vaccination with infection status, except in lung at day 21, for which an increase in relative abundance of Bacteroidetes and a decrease in relative abundance of Proteobacteria was observed (Fig. 5D). Remarkably, quantitative differences in the relative abundances of Cyanobacteria were associated with BCG vaccination at the gut level. An increase in Cyanobacteria relative abundance at day 7 was first associated with BCG vaccination (Fig. 5A) whereas a decrease in Cyanobacteria relative abundance was associated with BCG vaccination and M. tuberculosis infection at day 21. This decrement of Cyanobacteria abundance was also found at the lung level being associated with BCG vaccination and M. tuberculosis infection (Fig. 5C and D).

### Genus credibly associated with BCG vaccination and M. tuberculosis infection status and the gut–lung axis evolution.

**FIG 6:**
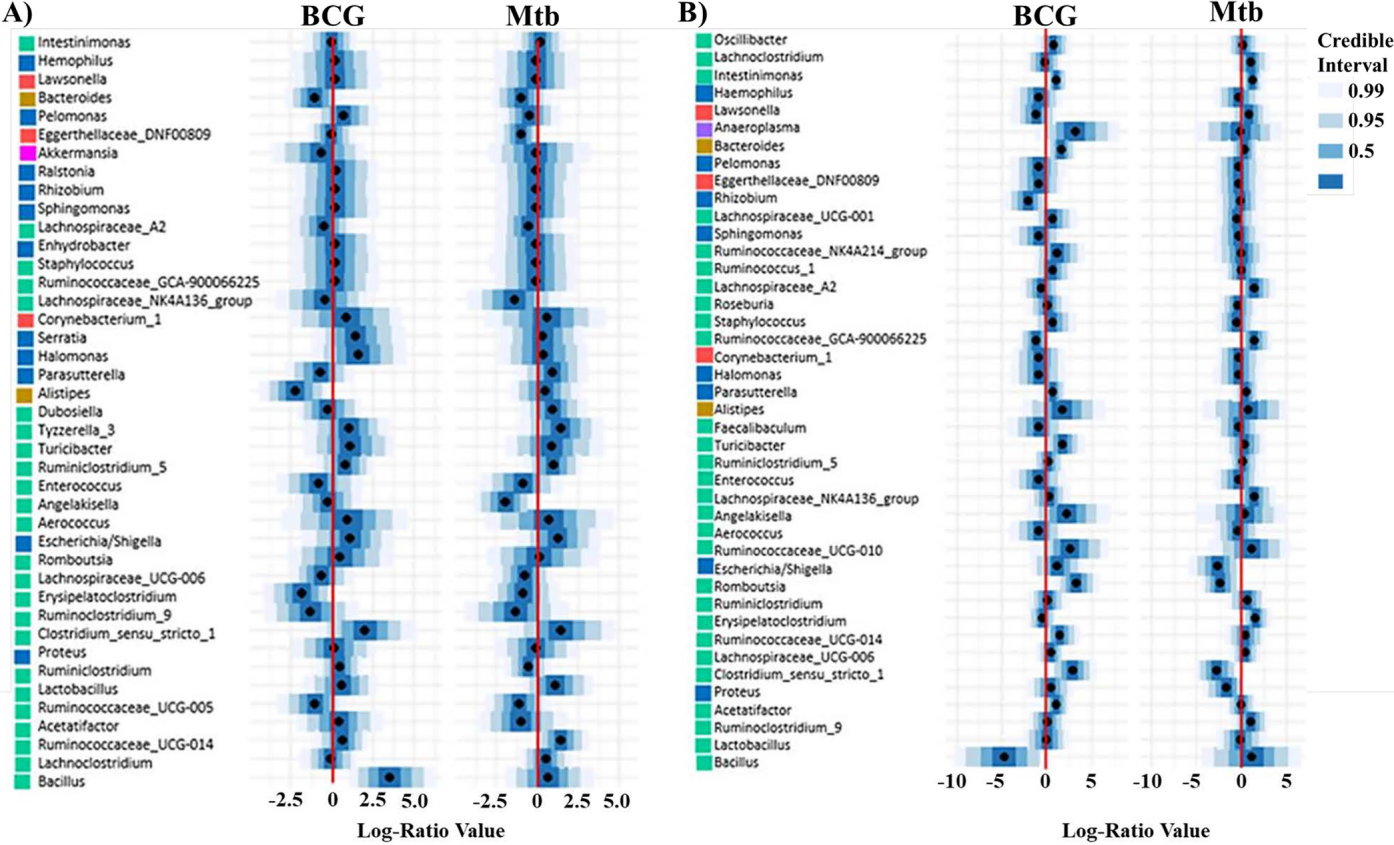
Gut genera found to be credibly associated with BCG vaccination and M. tuberculosis infection. Genera associated in at least one of our two conditions at day 7 (A) and day 21 (B) using a multivariate regression model.

**FIG 7:**
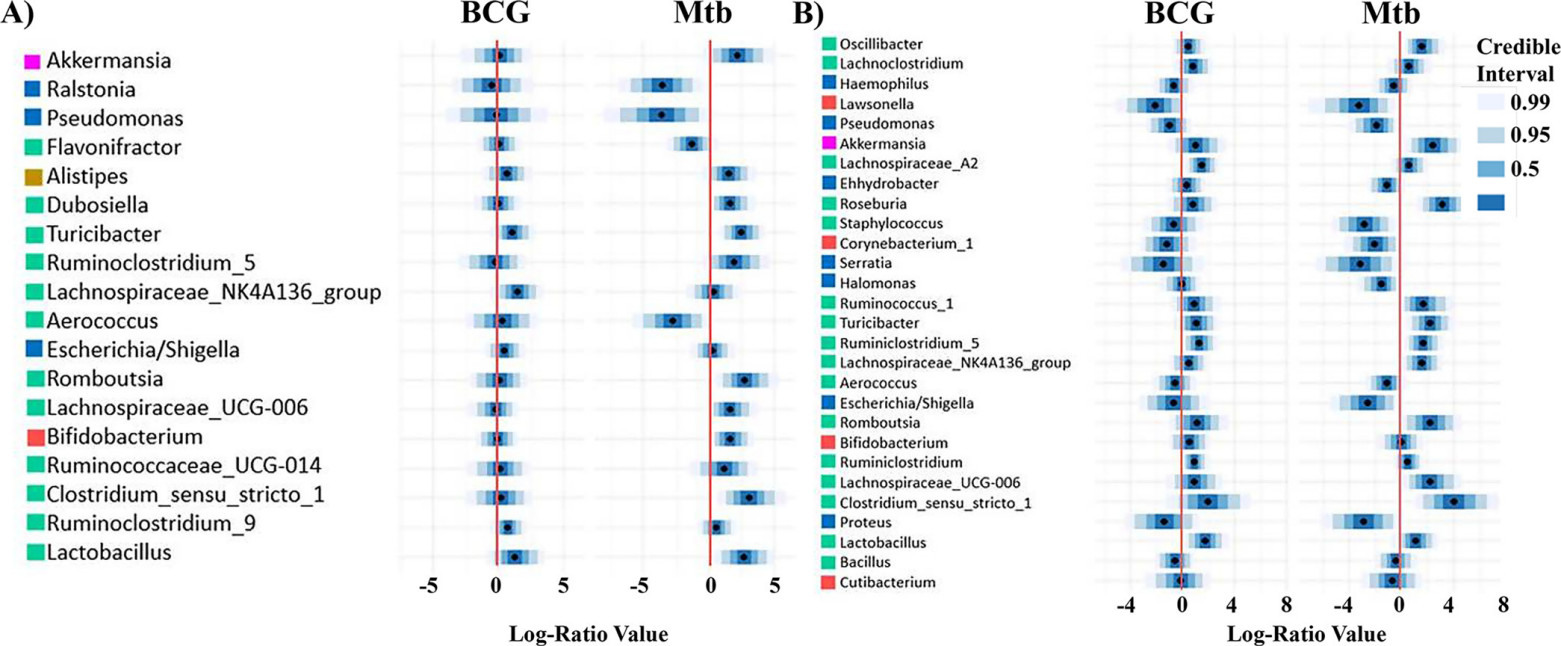
Lung genera found to be credibly associated with BCG vaccination and M. tuberculosis infection. Genera associated in at least one of our two conditions at day 7 (A) and day 21 (B) using a multivariate regression model.

Based on the same multivariate regression model applied to microbiota analysis at the genus level, numerous genera were correlated with BCG vaccination and M. tuberculosis infection (Fig. 6 and 7), with an increased genus number at day 21 compared with day 7 at the lung level (Fig. 7). Among them, several genera were selected as associated with non-zero effect.

At the gut level, BCG vaccination was associated with lower abundances of *Alistipes*, and *Erysipelatoclostridium*, along with a higher abundance of *Bacillus* at day 7 (Fig. 6A). At day 21, genus changes were more numerous as vaccination was associated with lower abundances of *Bacillus* and *Rhizobium* and with higher abundances of *Intestinimonas*, *Bacteroides*, *Turicibacter*, *Romboutsia*, and *Clostridium* (Fig. 6B).

M. tuberculosis infection was associated with lower abundances of Eggerthellaceae (DNF00809) and *Angelakisella*, and a higher abundance of Ruminococcaceae_UCG-014 at day 7 (Fig. 6A). At day 21, M. tuberculosis infection was associated with lower abundances of Escherichia/*Shigella*, *Romboutsia*, *Clostridium* and Proteus, and with higher abundances of *Intestinimonas* and *Erysipelatoclostridium* (Fig. 6B).

At the lung level, few genera were associated with vaccination status. Only a high abundance of Lachnospiraceae was associated with BCG vaccination at day 7 and confirmed at day 21 (Fig. 7A and B). At day 21, vaccination was also associated with higher abundances of *Lactobacillus* and *Ruminiclostridium* and with a lower abundance of *Lawsonella* (Fig. 7B).

Alternatively, M. tuberculosis infection was associated with a greater number of genera. At day 7, infection was associated with lower abundances of *Aerococcus*, *Flavonifractor*, *Ralstonia* and Pseudomonas, and with higher abundances of *Bifidobacterium*, *Alistipes*, *Clostridium*, *Dubosiella*, *Turicibacter*, *Lachnospiraceae*, *Lactobacillus*, *Romboutsia*, *Ruminiclostridium*, and *Akkermansia* (Fig. 7A). These associations were confirmed at day 21, except for the abundances of *Flavonifractor*, *Ralstonia*, *Bifidobacterium*, *Alistipes*, *Dubosiella*, and *Lactobacillus*. In addition, infection was associated with lower abundances of *Corynebacterium*, *Lawsonella*, Staphylococcus, Escherichia/*Shigella*, Proteus, *Serratia*, *Halomonas*, and *Enhydrobacter*, and with higher abundances of *Roseburia*, *Oscillibacter* and *Ruminococcus* (Fig. 7B).

Focusing on the gut–lung axis, our multivariate regression approaches allowed us to compare profile evolution of microbiota in gut and lung at days 7 and 21. More genera had modified relative abundances (not centered on log 0; Fig. 5) associated with BCG vaccination status at gut level compared with lung, especially at day 21 (Fig. 6B). Conversely, at the lung level, genera that had modified relative abundances associated with M. tuberculosis infection were numerous at both time points (Fig. 7), indicated that the host local response against infection impacted the whole microbial flora while the immune response after vaccination modified mainly the gut microbiota.

## DISCUSSION

Like the human lung, the most prevalent phyla found in the mice lung were Firmicutes, Proteobacteria, and Bacteroidetes. The prevalence of these three phyla have been previously reported (35–38). However, we observed high variability in the relative abundance at the phylum and genus levels based on the treatment group. As previously reported, we observed higher abundances of Escherichia*/Shigella* and *Alistipes* in gut microbiota of vaccinated and infected mice (39). Nevertheless, when comparing the two organs, the lung was the most impacted after BCG vaccination and M. tuberculosis infection. The establishment of a live microorganism through vaccination (attenuated) and/or infection may cause bias by observing an increase of bacterial diversity in the gut or lung. However, two ways to avoid this bias were included in this study. First, we included four groups of mice, including a control uninfected and unvaccinated group, and second, we analyzed the data considering the potential effect that the genus Mycobacterium may cause in our results.

**FIG 8:**
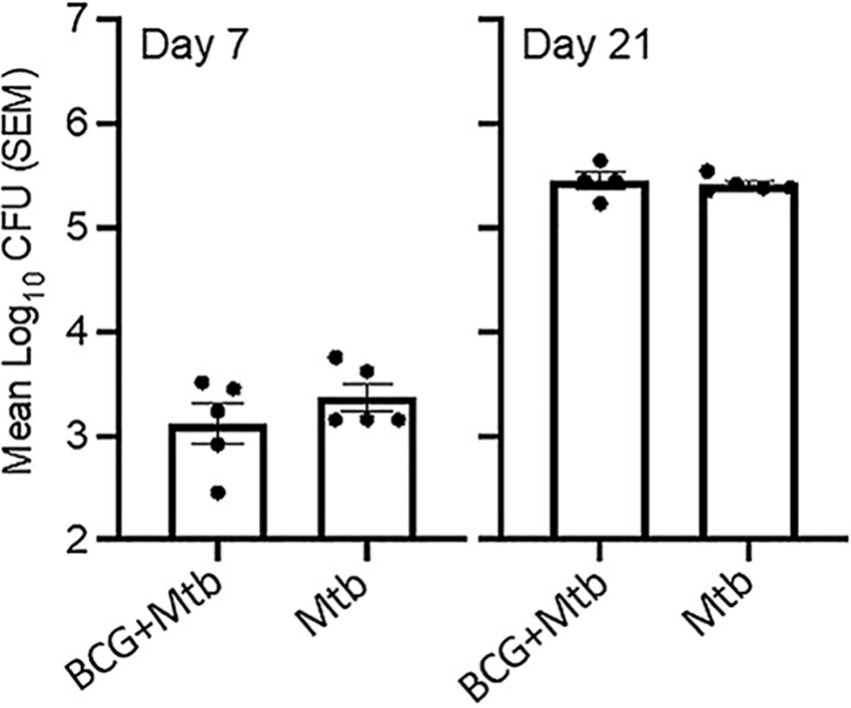
M. tuberculosis CFU obtained from lung. Lung from group 3 and 4 were assessed to determine the viable count of M. tuberculosis. No statistical differences were observed at day 7 or 21 postinfection.

The viable count of M. tuberculosis in the lung, did not show statistical differences between the BCG+Mtb and Mtb groups at day 7 (during the innate immune phase)- and 21 postinfection (at the beginning of the adaptive immune response) (Fig. 8). These results were supported by previous studies that shows that BCG vaccination provides better protection in mice challenged at 120 days postvaccination. Earlier infections after vaccination, as presented in our study, are characterized by the induction of CD4 IFN-γ^+^ TNF-α^+^ rather than CD4 IL-17^+^TNF-α^+^IL-2^+^, a memory cell phenotype critical for long-lasting control of M. tuberculosis (40–42).

It has been established that a healthy lung has low biomass and high bacterial diversity (43–45). However, our data show that BCG vaccination and M. tuberculosis infection increase bacterial alpha-diversity in the lungs. As observed in our results, some studies in mice have noted that an increased lung bacterial diversity may precede pathological outcomes in the lungs. For instance, Yang et al., 2019 (36) showed that an increased alpha-diversity precedes lung fibrosis via IL17R signaling in C57BL/6 mice. Another study by Cui et al., 2012, (46) showed that infected patients with symptomatic TB and under antimicrobial therapy had higher bacterial diversity and a more unique taxa compared with controls. Although infection and vaccination increase lung diversity, our results show that infection and vaccination mainly differ in the dominance of specific bacterial genera such as Pseudomonas, *Corynebacterium*, *Clostridium*, *Roseburia*, *Akkermansia*, and *Ruminococcus*.

Interestingly, Pseudomonas, an opportunistic pathogen associated with chronic lower respiratory infections, was found in lower abundance in the Mtb group. These results are remarkable because Pseudomonas is usually present as a primary pathogen in patients with lung chronic diseases such as CF, COPD, idiopathic pulmonary fibrosis, and bronchiectasis (38, 47–50). Pseudomonas can form biofilms during lung infection (48, 49). Additionally, they have been recognized as a critical bacterium associated with lung disease progression and severity, especially in CF (51, 52). Moreover, Pseudomonas was found more abundant in TB patients who were unsuccessfully treated and have had recurrent cases compared with other patient groups (53).

*Corynebacterium* was also observed in lower abundance in infected mice. This genus is normally present in the respiratory tract (54, 55), but with contradictory effects on the host. In BALB/c mice, the presence of this genus has been associated with resistance to viral and bacterial infections (54); however, in humans it has been associated with pneumonia (55, 56). Future analysis using shot gun metagenomics could determine the specific species associated with this change.

Conversely, *Clostridium*, *Roseburia*, *Akkermansia*, and *Ruminococcus* were observed in higher abundances in lung of infected mice. These genera have been associated with gut dysbiosis in human TB, but only *Clostridium* was increased in gut microbiota of TB patients compared with controls (22).

As previously reported during M. tuberculosis infection (57), Firmicutes were significantly elevated in lungs of infected mice compared with uninfected mice. Proteobacteria, which appears in higher proportion in the control group, decreased the abundance after infection and vaccination. Remarkably, a decrease in the relative abundance of Proteobacteria has been previously observed in lung aging studies. As we observed in the three treatment groups of this study, Lee et al. (58) reported an increase of Firmicutes and a decrease of Proteobacteria, which were associated with aging lung and reduced lung function. Thus, our results seem to indicate that lung dysbiosis induced by vaccination and infection may produce physiological changes and local oxidative stress in the lung, typically observed in elderly and TB patients (59). In agreement with our results, Firmicutes-dominated dysbiosis in the lung has been associated with the expression of pro-inflammatory genes in pulmonary leukocytes (17). These changes can also be correlated with local shifts observed by specific T and B cell immune responses previously reported in vaccinated and infected mice (60–63). Published data and our results suggest that changes in lung Firmicutes' relative abundance are observed as a signature of the typical innate and adaptive immune response to M. tuberculosis.

A metanalysis study performed by Eshetie and Soolingen (64) compared the lung microbiota of healthy and TB patients. This research suggested that Bacteroidetes are present in higher abundance in healthy controls (23.5% of abundance). In contrast, our results indicated that Bacteroidetes in the C57BL/6 mice model were present in very low abundance in lungs, and that BCG vaccination significantly increased the relative abundance of this phylum, as we observed in lungs of BCG *vs* control groups (Fig. 4D). However, these results need to be further documented since we also observed a significant increase of Bacteroidetes in lungs of Mtb group (Fig. 4E). Moreover, the same trend was observed when infected mice among BCG vaccinated mice were analyzed (Fig. 4F).

The Firmicutes/Bacteroidetes (F/B) ratio is essential to maintaining the intestinal homeostasis. For instance, an increased or decreased ratio has been associated with obesity or inflammatory bowel disease, respectively (65–67). Our study showed that control, BCG, and BCG+Mtb groups had a more similar intestine F/B ratio, compared with Mtb group (control: 2.13 ± 0.77; BCG: 2.96 ± 0.63; BCG+Mtb: 3.18 ± 0.78; Mtb: 1.56 ± 0.44).

Our study showed that the gut and lung have direct bidirectional communication with microbiota changes as early as 7 days post-M. tuberculosis infection. Using a multivariate regression approach, we were able to show that microbiota profile evolution was more pronounced after BCG vaccination at the gut, and after M. tuberculosis infection at the lung (Fig. 6 and 7). This indicates that the host local immune response against infection impacted the whole microbial flora, whereas the immune response after vaccination modified mainly the gut microbiota. Several of the modified taxa that we identified in this study have been previously described as host immune modulators: For example, Bacteroides, a phylum increased in gut microbiota of vaccinated mice at 21 days (Fig. 6B), produces polysaccharides that mediate mucosal tolerance via upregulation of regulatory T (Treg) cells (68). In addition, *Lactobacillus* which can modulate adaptive and innate immune responses via direct binding to pattern recognition receptors (69), was found in higher abundance in gut and lung of vaccinated and infected mice (Fig. 6 and 7). As observed in previous experiments, possible bidirectional gut–lung communications may include (i) direct migration of innate lymphoid and Th17 cells (70, 71), (ii) elicitation of lung Interferon through the microbial metabolite desamirotyrosine produced in the intestine (72), and (iii) development of immune cells in the bone marrow through the effect of unmetabolized short-chain fatty acids (SCFAs) derived from the metabolism of dietary fibers (73, 74).

The effect of vaccination on local or systemic microbiota and on the immune response against infection has not been fully addressed. Current efforts have been focused on understanding how the stablished of microbiota may affect the immune response of vaccination. For instance, some reports show that there is a poor immune response after the administration of oral polio or cholera vaccines in children of developing countries, mainly due to lower oral and hand hygiene from early life (75–77). On the contrary, our study aimed to investigate the effect of vaccination and infection on subsequent changes of host microbiota, therefore, phyla or genus can be potentially targeted as markers of immune response or disease progression.

In a near future, microbial therapies could help in treating patients suffering from TB or in improving BCG vaccination response, nonetheless further studies focused on the gut–lung axis are needed.

## CONCLUSIONS

We demonstrated for the first time that lung and gut dysbiosis are induced via BCG vaccination through the subcutaneous route. The specific microbiota interactions observed in the gut and lung after M. tuberculosis infection must be further studied especially through shot gun metagenomics. Well-designed studies on the M. tuberculosis long-term effects could determine specific bacterial species involved in the disease progression and could also determine whether treatment with specific microbiota, such as fecal transplantation, may reduce severity to M. tuberculosis infection.

## MATERIALS AND METHODS

### Study design and sample collection.

**FIG 9:**
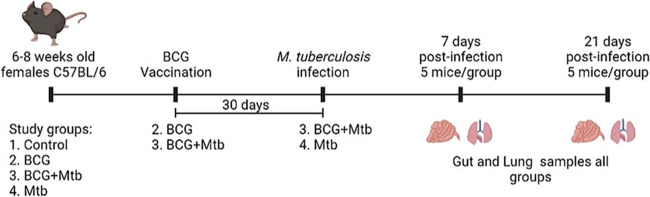
Timeline of vaccination, infection, and sampling. Timeline of the vaccination, infection, and sampling of the study groups; 1. control, 2. BCG vaccinated (BCG), 3. BCG vaccinated and M. tuberculosis-infected (BCG+Mtb), 4. M. tuberculosis-infected (Mtb). All study groups consisted of 10 mice/group. The dose of the BCG vaccine was 5 × 10^5^ CFU/mL. The M. Tuberculosis inoculum was 2 × 10^6^ CFU/mL.

M. bovis BCG Pasteur (TMC#1011) and M. tuberculosis H37Rv (TMC#102) were used in this study. Forty 6–8 weeks old female C57BL/6 mice (Jackson Laboratories, Bar Harbor, ME) formed the study groups. Mice were divided into four groups (Fig. 9): (i) control (control, *n* = 10), (ii) BCG vaccinated (BCG, *n* = 10), (iii) BCG vaccinated and M. tuberculosis-infected (BCG+Mtb, *n* = 10), and (iv) M. tuberculosis-infected without vaccination (Mtb, *n* = 10). All the mice were housed in BSL-3 throughout the duration of the study at Colorado State University and were all treated identically, except for the vaccination and/or infection. All mouse cage changes were performed at the same time in a Biosafety cabinet. Mice from groups BCG and BCG+Mtb were vaccinated with BCG Pasteur via subcutaneous administration in the left flank with a dose of 5 × 10^5^ CFU/mL. Thirty days after experiment initiation, mice from groups BCG+Mtb and Mtb were infected with M. tuberculosis H37Rv with 2 × 10^6^ CFU/mL to deliver 50–100 CFU using the Inhalation Exposure System (Glas-Col, Terre Haute, IN). The inoculum was plated on 7H11 agar to ensure the correct number of viable organisms was placed into the nebulizer. The exposure machine was kept in a BSL-3 room near the room in which the mice were kept, and therefore, the airflow into the rooms was similar throughout the BSL-3 wing of the animal facility. Five mice were sacrificed on the day of infection to check the number of CFU implanted into the lung. At 7 and 21 days postinfection, five mice per group were sacrificed (Fig. 9). The left lung lobe and a portion of the small intestine of three mice per group were aseptically removed for DNA extractions. In addition, the lung from groups BCG+Mtb and Mtb were aseptically removed for viable M. tuberculosis CFU counting. Removed lungs were homogenized in sterile saline before being diluted and plated on 7H11 agar. Plates were incubated at 37°C for 14 days. The resulting CFU was converted into Log_10_ for analysis. One mouse from group BCG+Mtb (day 21 postinfection) was excluded from the analysis due to ulcerative dermatitis.

The Institutional Animal Care and Use Committee at Colorado State University reviewed and approved all animal experiments (Protocol ID: 16-6369A).

### DNA extraction and library preparation.

According to the manufacturer's instructions, DNA was extracted from approximately 25 mg of lung and intestine samples using the DNeasy blood and tissue kit (Qiagen, Hilden, Germany). Initial DNA concentration and quality were assessed using Nanodrop (Invitrogen, California, USA) and stored at −80°C. Two successive PCR tests were performed to amplify each target regions and attach adapters and indexes. For the PCR1, the DNA was standardized to 20 ng/μL and checked in agarose gel to verify the amplicon size and the absence of unspecific bands. The microbial diversity and taxonomic composition of samples were assessed by using the V3-V4 region of the bacterial 16S rRNA gene. The primers used to amplify the V3-V4 were 16S-Forward (TACGGRAGGCAGCAG) and 16S-Reverse (CTACCNGGGTATCTAAT), as previously described (78). The included controls were the ZymoBIOMICS Microbial DNA Community Standard (mock community) and a no-template negative control, which were both processed alongside the mice samples, to validate the experimental procedures. Briefly, PCR amplification was performed by using barcoded primers (at a final concentration of 0.2 μM) with an annealing temperature of 50°C for 30 cycles and mixed in equimolar amounts to be sequenced. PCR1 amplicons were purified to eliminate primers and primer dimers. Briefly, an adjusted volume of magnetic beads was added to each plaque well, mixed, and centrifuged 2 min at 1,800 rpm. The plaque was incubated for 5 min at room temperature and placed in DynaMag-96 Side Skirted Magnet (Invitrogen) for 2 min. After incubation, the supernatant was eliminated, and two consecutive washes with 70% EtOH were performed while keeping the plaque in the magnet. After EtOH washes, the plaque was let dry for 5 min and removed from the magnet. 25 μL of 10 mM Tris pH 8.5 was added to each well, mixed, centrifuged for 2 min at 1,800 rpm, and incubated at room temperature for 2 min. The plaque was then placed in the magnet for 2 min, and 20 μL of the clear supernatant was transferred to a new PCR plaque. After purification, the master mix preparation was performed for PCR2 to add indexes and adapters. PCR1 products were diluted 1:10, and indexes were added at 5 μM. PCR2 products were quantified to pool the samples in equimolar. The library's size was verified with Tapestation, normalized, and pooled before sequencing.

### Sequencing and bioinformatic analysis.

Next-generation sequencing was performed by using 250-bp paired-end technology on the MiSeq platform (Illumina, San Diego, CA) with V3 chemistry at the PGTB platform of Bordeaux University. The bacterial reads were demultiplexed, then 16S primers were removed using CutAdapt, with no mismatch allowed within the primer sequences. All samples were processed through the DADA2 pipeline in R (version 4.0.3) for quality filtering and trimming, dereplication, and merging of paired ends read (79–81). The distinct ASV table was constructed, and bacterial taxonomy was assigned from Silva database (release 138). Mock community was used to avoid a non-efficient sequencing experiment, and negative controls to identify and remove potential reagent contaminants with microDecon R package (82). ASVs present in less than three samples were removed (34). The final read counts were 285,256 (Mean of 11,886 ± 10,561) at the intestine site and 35,179 (Mean of 1,466 ± 3,495) at the lung site.

### Statistical analysis.

A nonparametric Wilcoxon-Mann-Whitney test was used to compare quantitative variables among groups. Statistical analyses were performed with the RStudio program (version 4.0 for Windows). A *P*-value < 0.05 was considered indicative of statistical significance.

Alpha- and beta-diversity indexes were assessed using ASV occurrence counts and a multidimensional Scaling (MDS) ordination method with Bray-Curtis distance metric implemented by R package 'Phyloseq' for each organ (gut and lung). To compute beta-diversity, the ASVs table was first rarefied (using phyloseq’s rarefy_even_depth function) at a minimum sequence depth. Between sample beta-diversity differences (measured using Bray Curtis dissimilarity) were tested using a PERMANOVA from vegan R package with 10,000 permutations, while accounting for individual identity as a covariate.

DESeq2 (83) was used to perform two-class testing for differential relative abundance. Paired tests (by subject) were used when comparing gut and lung microbiota diversities of groups, as follow: (i) vaccination effect was assessed by comparing uninfected mice (control *vs* BCG group) independently of the day of sacrifice (7 or 21), and (ii) M. tuberculosis infection effect was assessed by comparing infected mice without vaccination on one side (control *vs* Mtb group) independently the day of sacrifice (7 or 21), and infected and vaccinated mice on the other side (Mtb versus BCG+Mtb) independently of the day of sacrifice (7 or 21).

To associate microbial composition with either BCG vaccination or M. tuberculosis infection effects, we used Bayesian multinomial logistic-normal linear regression, implemented in the R package stray as the function pebble (34, 84). As previously described (34, 84), we chose this method because it considers uncertainty due to counting and compositional data usually known in targeted metagenomics. In addition, Holmes et al. (34) proposed to transform results into centered log-ratio coordinates for interpretation, based on theory from compositional data analysis and credible intervals and figures.

### Data availability.

The fastq files and metadata are available in the Sequence Reads Archive (SRA). Accession number PRJNA768241.
